# The role of dentate status and dental caries on diabetes-related complications: a hospital-based cross-sectional study

**DOI:** 10.25122/jml-2024-0405

**Published:** 2024-12

**Authors:** Sunithi Thearawiboon, Chanapong Rojanaworarit

**Affiliations:** 1 Dental Department, Prachathipat Hospital, Pathum Thani, Thailand; 2 Department of Population Health, School of Health Sciences, Hofstra University, Hempstead, New York, USA

**Keywords:** dental caries, edentulism, diabetes mellitus, retinopathy, diabetic foot, chronic kidney disease

## Abstract

This study explored the role of dentate status and dental caries on diabetes-related complications among patients with type 2 diabetes mellitus (T2DM). A hospital-based cross-sectional design was applied to collect data on diabetic patients attending integrated services for non-communicable diseases and oral health at a public hospital in Thailand. Diabetic complication outcomes included diabetic eye and foot complications and chronic kidney disease (CKD). The main independent variable of dentate status and dental caries was classified into three categories: dentate and caries-free, dentate with caries, and edentulous. The relationships were evaluated through epidemiological models depicted by directed acyclic graphs (DAGs). Multivariable Poisson regression with robust standard errors was applied to estimate prevalence ratios (PR) according to DAGs. Among 438 patients with T2DM, 62.8% were women, and an average age was 63.6 years. Most patients were dentate with dental caries (70.1%), and 8.2% were edentulous. Prevalence of diabetic foot complications and CKD were 37.1% and 10.1%. Six patients had eye complications. Regarding CKD outcome, PR estimates from univariable and multivariable models were 0.94–1.12 for the dentate with caries group and 1.67–2.31 for the edentulous group, all with non-significant *P* values. Regarding foot complication outcome, PR estimates were 1.10–1.12 for the dentate with caries group and 1.26–1.37 for the edentulous group, all with non-significant *P* values. Though not statistically significant, the magnitude and direction of PR suggested a possible hypothesis that, among patients with T2DM, edentulism might be related to a higher prevalence of CKD and diabetic foot complications as compared to being dentate and caries-free.

## INTRODUCTION

The etiologic mechanisms linking dental caries to diabetes mellitus involve hyperglycemia, salivary alterations, and a compromised immune response [1-3]. Hyperglycemia or elevated blood sugar levels in diabetes mellitus can raise salivary sugar levels and create a favorable oral environment rich in nutrients for cariogenic bacteria such as *Streptococcus mutans* [4,5]. Regarding salivary alteration, diabetes mellitus can reduce the salivary flow rate and alter salivary buffering capacity [3]. The decrease in salivary flow rate or hyposalivation results in diminished natural cleansing action of saliva that normally removes food debris and bacteria from the teeth [6]. The altered salivary buffering capacity impacts the neutralization of acids that induce demineralization of dental hard tissues [3]. Diabetes mellitus can also impair the host immune system, resulting in a compromised defense mechanism that yields the progression of dental caries [2]. Conversely, dental caries may adversely affect the systemic condition of diabetes mellitus by contributing to systemic inflammation [7], which is associated with a decrease in insulin sensitivity and complicates the control of blood sugar levels [8,9].

Edentulism, defined as the complete loss of all teeth [10], has been identified as a condition associated with diabetes mellitus, as evidenced by epidemiological studies [11-13]. The prevalence of edentulism among individuals with diabetes mellitus is approximately double that of those without diabetes (28% versus 14%) [11]. In the US, diabetes was associated with one in every five cases of edentulism [11]. Similarly, studies in 40 low- and middle-income countries revealed that adults with diabetes mellitus had significantly higher odds of being edentulous compared to their non-diabetic counterparts [12]. The implications of edentulism extend beyond oral health, with evidence suggesting a broader impact on general health. For instance, a study demonstrated that individuals with both diabetes and edentulism had poorer overall health compared to those with diabetes alone, with declines in mental processing, perceived stress, sleep quality, and energy levels [12]. This suggested a possible role of edentulism and diabetes as potential comorbid factors affecting general health [12,14]. In addition, edentulism may also adversely impact blood sugar levels among patients with diabetes. In a comparative study, diabetic individuals with edentulism had significantly higher blood sugar levels and greater odds of hyperglycemia than those with moderate to severe periodontitis or no/mild periodontitis [15].

Extensive evidence has linked the localized oral conditions of dental caries and edentulism to the systemic disease of diabetes mellitus. Furthermore, biological plausibility suggests that these oral conditions may contribute to systemic inflammation [7] and hyperglycemia [8,9,15], both of which are critical factors in developing diabetes-related complications. However, the potential connection between these oral conditions and diabetes-related complications—such as diabetic retinopathy, diabetic foot ulcers, and chronic kidney disease—remains largely unexplored. These complications often arise after prolonged periods of chronic inflammation and hyperglycemia in patients with diabetes. Investigating this connection may provide clues as to whether dental caries and edentulism can be comorbid factors prognosticating diabetic complications. In addition, based on the causal inference approach in epidemiology to identify valid exposure-outcome relationships, directed acyclic graphs (DAG) can be applied to determine the different roles of explanatory variables–such as intermediate and confounding variables–that are involved in the relationship between the main exposure variables of dental caries and edentulism and the outcomes of diabetic complications [16,17].

Therefore, this study aimed to explore the role of dentate status and dental caries in diabetes-related complications among patients with type 2 diabetes mellitus (T2DM). The use of DAG was incorporated to improve the validity of the measures indicating the relationship between dentate status, dental caries, and diabetic complication outcomes.

## MATERIAL AND METHODS

### Study design and patients

A hospital-based cross-sectional study design was applied to collect clinical data of all patients with T2DM attending integrated non-communicable diseases (NCDs) and dental care services at Prachathipat Hospital, a public hospital providing primary and secondary medical care in Prachathipat Subdistrict, Thanyaburi District, Pathum Thani Province, Thailand between October 2022 and June 2023.

### Outcome measurement

Outcomes of diabetes-related complications included diabetic eye complications, diabetic foot complications, and chronic kidney disease (CKD). The diabetic eye complication was determined by the presence of non-proliferative diabetic retinopathy (NPDR) using fundus photography during an ophthalmologic examination. Diabetic foot complications were determined by the physical therapist through a clinical examination of the foot and a mono-filament test. The presence of at least one of the following clinical findings confirmed the foot complication: foot deformity, foot numbness, weakening or loss of posterior tibial or dorsalis pedis pulses, non-response to pressure applied during mono-filament testing, foot ulcer, and foot amputation. The diagnosis of CKD was determined based on the physician's diagnostic record and supported by at least one of the following laboratory findings: albumin-to-creatinine ratio ≥ 30 mg/g, urine protein-to-creatinine ratio ≥ 150 mg/g, and estimated glomerular filtration rate (eGFR) < 60 ml/min/1.73 m^2^.

### Exposure measurement

The major exposure variable of dentate status and dental caries was categorized into three categories: dentate and caries-free, dentate with caries, and edentulous. This categorization was applied to reflect three real-world oral statuses of patients and allow comparisons of the effects of having dental caries and edentulism to the same referent category of dentate and caries-free, which have scarcely been investigated in previous literature. Other clinical variables included sex, age, body mass index (BMI), diabetic control, hypertension, myocardial infarction (MI), heart failure (HF), cerebrovascular disease, and cancer.

### Data source

Demographic and clinical data of all patients were retrieved from the hospital’s electronic database. All required data were ascertained to be recorded on the same visit. Only data from the patient’s latest visit within the study period were used for analysis.

### Statistical analysis

Descriptive statistics were used to summarize the characteristics of the study patients. Independent samples *t*-test, one-way analysis of variance (ANOVA), and Bonferroni post hoc test were applied to compare means between groups where applicable. An exact probability test was used to compare proportions across groups.

The exposure-outcome relationship for each diabetic complication outcome was evaluated through several epidemiological models depicted by a directed acyclic graph (DAG). Univariable Poisson regression with robust standard errors was undertaken to estimate the crude prevalence ratio (PR) and 95% confidence interval (CI) for the relationship between each explanatory variable and each diabetic complication outcome. This univariable analysis facilitated the selection of explanatory variables for subsequent multivariable analysis. A DAG was developed for each outcome to visualize the relationships between selected explanatory variables included in the multivariable analysis. Multivariable Poisson regression with robust standard errors was sequentially applied to estimate adjusted PR and 95% CI according to models in DAG. To prevent sparse data bias in the estimates obtained from the regression analyses, any diabetic complication outcome with a low prevalence that could result in the lack of case numbers for several combinations of exposure and outcome levels was omitted from the regression analyses [18]. Apart from the PR estimation, an additional analysis of the magnitude of confounding indicating the percent difference between the crude and adjusted estimates of PR was also applied to determine the extent to which potential confounding variables influenced the observed relationship between the main exposure of dentate status and dental caries and each diabetic complication outcome. The formula for the percent difference of [(PR_crude_– PR_adjusted_)/PR_adjusted_] ×100 was applied, and the percent difference of ≥ 10% was used to conclude that there was confounding.

## RESULTS

Among the 438 patients with T2DM in this study, 70.1% were dentate with caries, and 8.2% were edentulous. Most patients were women (62.8%), and the average age of all patients was 63.6 years. A one-way ANOVA comparing the mean ages across the three dental status categories showed a significant difference (P < 0.001). The Bonferroni post hoc test identified that the average age of edentulous patients was significantly higher than patients in the other two dental categories (P < 0.001 for both comparisons). The overall average number of remaining teeth was 18.1. About 52.5% of all patients had ≥ 20 remaining teeth. The average number of remaining teeth in dentate patients with dental caries was not significantly higher than that of the dentate and caries-free patients (P = 0.074). Slightly more than half of all patients (53.6%) were overweight or obese. However, average BMI values across the three dental categories were not significantly different (P = 0.065). Nearly half of the patients (49.3%) had uncontrolled diabetes. The most common comorbidity was hypertension (93.8%). About 37.1% and 10.1% had diabetic foot complications and CKD. Only 6 patients (1.4%) had eye complications, indicating the relatively rare occurrence of this complication among patients with T2DM. (Table 1)

**Table 1:** Characteristics of patients with diabetes by dentate status and presence of dental caries

Characteristics	Total *n* (%)^†^	Dentate status and dental caries			*P* value
		Dentate and caries-free	Dentate with caries	Edentulous	
		*n* (%)^‡^	*n* (%)^‡^	*n* (%)^‡^	
**Overall**	**438**	**95 (21.7)**	**307 (70.1)**	**36 (8.2)**	
**Sex**					
Female	275 (62.8)	67 (24.4)	182 (66.1)	26 (9.5)	0.073^*^
Male	163 (37.2)	28 (17.2)	125 (76.7)	10 (6.1)	
**Age**					
Mean ± SD	63.6 ± 10.1	61.6 ± 10.6	63.1 ± 9.5	73.6 ± 8.3	<0.001^**^
Min. – Max.	35 – 87	35 – 86	36 – 83	59 – 87	
Age ≤ 59	141 (32.2)	39 (27.7)	101 (71.6)	1 (0.7)	
Age ≥ 60	297 (67.8)	56 (18.9)	206 (69.3)	35 (11.8)	
**Number of remaining teeth**					
Mean ± SD	18.1 ± 9.62	18.4 ± 9.55	20.14 ± 7.83	N/A	0.074^***^
Min. – Max.	0 – 32	2 – 32	1 – 32	N/A	
< 20 teeth	208 (47.5)	45 (21.6)	127 (61.1)	36 (17.3)	
≥ 20 teeth	230 (52.5)	50 (21.7)	180 (78.3)	0 (0)	
**BMI**					
Mean ± SD	26.0 ± 5.3	26.7 ± 5.7	25.9 ± 5.2	24.3 ± 4.4	0.065^**^
Min. – Max.	15.8 – 45.9	17.5 – 43.7	15.8 – 45.9	15.8 – 33.7	
< 18.5 (Underweight)	23 (5.3)	3 (13.0)	17 (74.0)	3 (13.0)	
18.5 – 24.9 (Normal)	180 (41.1)	37 (20.6)	125 (69.4)	18 (10.0)	
25.0 – 29.9 (Overweight)	148 (33.7)	33 (22.3)	104 (70.3)	11 (7.4)	
≥ 30 (Obese)	87 (19.9)	22 (25.3)	61 (70.1)	4 (4.6)	
**Diabetic control**					
Well-controlled	222 (50.7)	48 (21.6)	156 (70.3)	18 (8.1)	>0.999^*^
Uncontrolled	216 (49.3)	47 (21.8)	151 (69.9)	18 (8.3)	
**Hypertension**					
No	27 (6.2)	6 (22.2)	20 (74.1)	1 (3.7)	0.848^*^
Yes	410 (93.8)	88 (21.5)	287 (70.0)	35 (8.5)	
**Myocardial infarction**					
No	432 (98.6)	94 (21.8)	302 (69.9)	36 (8.3)	>0.999^*^
Yes	6 (1.4)	1 (16.7)	5 (83.3)	0 (0)	
**Heart failure**					
No	435 (99.3)	95 (21.8)	305 (70.1)	35 (8.1)	0.336^*^
Yes	3 (0.7)	0 (0)	2 (66.7)	1 (33.3)	
**Cerebrovascular disease**					
No	436 (99.5)	94 (21.6)	307 (70.4)	35 (8.0)	0.042^*^
Yes	2 (0.5)	1 (50.0)	0 (0)	1 (50.0)	
**Cancer**					
No	436 (99.5)	94 (21.6)	306 (70.1)	36 (8.3)	0.509^*^
Yes	2 (0.5)	1 (50.0)	1 (50.0)	0 (0)	
**Diabetic complications**					
No	251 (57.4)	58 (23.1)	176 (70.1)	17 (6.8)	0.361^*^
Yes	186 (42.6)	37 (19.9)	130 (69.9)	19 (10.2)	
**Eye complication**					
No	432 (98.6)	92 (21.3)	304 (70.4)	36 (8.3)	0.196^*^
Yes	6 (1.4)	3 (50.0)	3 (50.0)	0 (0)	
**Chronic kidney disease**					
No	394 (89.9)	87 (22.1)	278 (70.5)	29 (7.4)	0.144^*^
Yes	44 (10.1)	8 (18.2)	29 (65.9)	7 (15.9)	
**Foot complication**					
No	275 (62.9)	63 (22.9)	192 (69.8)	20 (7.3)	0.529^*^
Yes	162 (37.1)	32 (19.8)	114 (70.3)	16 (9.9)	

SD, Standard deviation; Min., Minimum; Max., Maximum; N/A, Not applicable

^†^ Column percentage; ^‡^ Row percentage

* Exact probability test, ** One-way analysis of variance, *** Independent samples *t*-test

Findings from the univariable analyses are presented in Table 2. The primary exposure variable, dentate status and dental caries, was categorized as dentate and caries free (referent category), dentate with caries, and edentulous. Comparisons were made using the dentate and caries-free group as the reference. Due to the low prevalence of diabetic eye complications (1.4%) among the study population, regression analysis for this outcome was excluded. This decision was made to avoid imprecise regression estimates that could result from sparse data bias [18].

**Table 2:** Univariable analyses of patients’ characteristics and outcomes of chronic kidney disease and diabetic foot complication

Characteristics	Chronic kidney disease		Univariable analysis for chronic kidney disease			Diabetic foot complication		Univariable analysis for diabetic foot complication		
	Yes	No	PR^‡^	95% CI	*P* value	Yes	No	PR^‡^	95% CI	*P* value
	*n* (%)^†^	*n* (%)^†^				*n* (%)^†^	*n* (%)^†^			
**Overall**	**44 (10.1)**	**394 (89.9)**		**-**		**162 (37.1)**	**275 (62.9)**		**-**	
**Main exposure**										
**Dentate status and dental caries**										
Dentate and caries-free	8 (8.4)	87 (91.6)		Reference		32 (33.7)	63 (66.3)		Reference	
Dentate with caries	29 (9.5)	278 (90.5)	1.12	0.53, 2.37	0.764	114 (37.3)	192 (62.7)	1.11	0.80, 1.52	0.534
Edentulous	7 (19.4)	29 (80.6)	2.31	0.90, 5.91	0.081	16 (44.4)	20 (55.6)	1.32	0.83, 2.09	0.240
**Covariates**										
**Sex**										
Female	20 (7.3)	255 (92.7)		Reference		103 (37.5)	172 (62.5)		Reference	
Male	24 (14.7)	139 (85.3)	2.02	1.15, 3.55	0.014	59 (36.4)	103 (63.6)	0.97	0.75, 1.25	0.829
**Age**										
< 60 years	5 (3.6)	136 (96.4)		Reference		47 (33.3)	94 (66.7)		Reference	
≥ 60 years	39 (13.1)	258 (86.9)	3.70	1.49, 9.20	0.005	115 (38.9)	181 (61.1)	1.16	0.89, 1.53	0.273
**BMI**										
18.5 – 24.9 (Normal weight)	21 (11.7)	159 (88.3)		Reference		67 (37.4)	112 (62.6)		Reference	
< 18.5 (Underweight)	2 (8.7)	21 (91.3)	0.75	0.19, 3.00	0.678	6 (26.1)	17 (73.9)	0.70	0.34, 1.42	0.322
25.0 – 29.9 (Overweight)	13 (8.8)	135 (91.2)	0.75	0.39, 1.45	0.397	51 (34.5)	97 (65.5)	0.92	0.69, 1.23	0.579
≥ 30 (Obese)	8 (9.2)	79 (90.8)	0.79	0.36, 1.71	0.547	38 (43.7)	49 (56.3)	1.17	0.86, 1.58	0.321
**Hypertension**										
No	0 (0)	27 (100.0)		N/A		6 (22.2)	21 (77.8)		Reference	
Yes	44 (10.7)	366 (89.3)		N/A		156 (38.1)	253 (61.9)	1.72	0.84, 3.52	0.140
**Myocardial infarction**										
No	43 (10.0)	389 (90.0)		Reference		161 (37.4)	270 (62.6)		Reference	
Yes	1 (16.7)	5 (83.3)	1.67	0.27, 10.27	0.577	1 (16.7)	5 (83.3)	0.45	0.07, 2.69	0.378
**Heart failure**										
No	43 (9.9)	392 (90.1)		Reference		162 (37.3)	272 (62.7)		N/A	
Yes	1 (33.3)	2 (66.7)	3.37	0.66, 17.16	0.143	0 (0)	3 (100.0)		N/A	
**Cerebrovascular disease**										
No	44 (10.1)	392 (89.9)		N/A		162 (37.2)	273 (62.8)		N/A	
Yes	0 (0)	2 (100.0)		N/A		0 (0)	2 (100.0)		N/A	
**Cancer**										
No	44 (10.1)	392 (89.9)		N/A		162 (37.2)	273 (62.8)		N/A	
Yes	0 (0)	2 (100.0)		N/A		0 (0)	2 (100.0)		N/A	

PR, prevalence ratio; CI, confidence interval; N/A, Not applicable.

† Percentage by row. ‡ Prevalence ratio estimated by Poisson regression with robust standard errors.

For the CKD outcome, crude PR estimates for dentate patients with caries and edentulous patients were 1.12 and 2.31. Crude PR estimates for variables including sex, age, BMI, myocardial infarction (MI), and heart failure (HF) were also obtained. Nonetheless, only 1 out of 6 patients with MI and 1 out of 3 patients with HF had CKD outcome. Therefore, MI and HF were not further selected for the subsequent multivariable analyses because the obtained crude PR estimates of 1.67 for MI and 3.37 for HF were calculated based on sparse data, and this could potentially result in the imprecise estimates obtained from multivariable models [18]. Additionally, variables such as hypertension, cerebrovascular disease, and cancer were excluded from multivariable analyses because the absence of CKD cases in certain cells of the contingency tables made it impossible to calculate PR. (Table 2)

For diabetic foot complication outcome, crude PR estimates for dentate patients with caries and edentulous patients were 1.11 and 1.32. Covariates of sex, age, BMI, hypertension, and MI also provided crude PR estimates. However, only 1 out of 6 patients with MI had diabetic foot outcomes. Therefore, MI was not selected for multivariable analyses. Variables, including HF, cerebrovascular disease, and cancer, were excluded from multivariable analyses due to no diabetic foot outcome among patients with these comorbidities. (Table 2)

**Figure 1:**
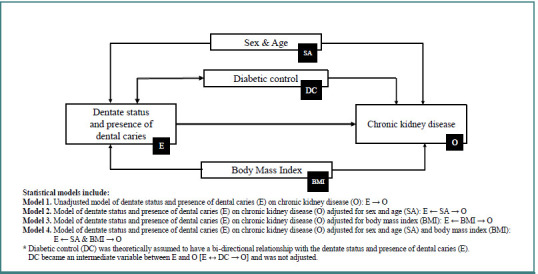
Directed acyclic graphs for analysis of the relationship between dentate status and presence of dental caries and chronic kidney disease outcome

**Figure 2:**
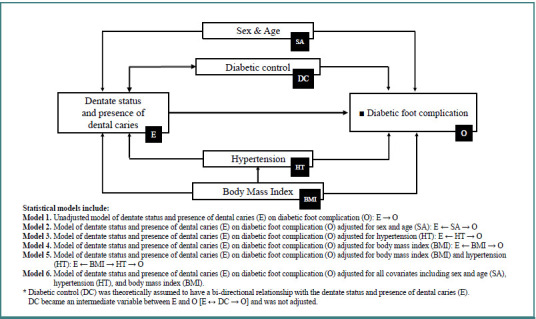
Directed acyclic graphs for analysis of the relationship between dentate status and presence of dental caries and outcome of diabetic foot complication

Based on the variable selection process for multivariable analyses, DAGs were created for CKD and diabetic foot outcomes and are presented in Figures 1 and 2. According to both figures, the diabetic control factor was theoretically assumed to have a bi-directional relationship with the main exposure factor of dentate status and dental caries. Diabetic control was an intermediate variable between the main exposure and diabetic complication outcomes and was not adjusted in the multivariable analyses.

Tables 3 and 4 present results from multivariable analyses sequentially executed according to the DAGs for CKD and diabetic foot outcomes. For CKD (Table 3), PR estimates across three multivariable models adjusting for different sets of potential confounding factors (Models 2–4) ranged from 0.94 to 1.12 for dentate patients with caries and 1.67 to 2.27 for edentulous patients, with all *P* values being non-significant. In the group of dentate patients with caries, the adjusted PR estimates of 0.94 obtained from Models 2 and 4 markedly differed from the crude PR estimate of 1.12 in Model 1. The magnitude of confounding was calculated as [(1.12-0.94)/0.94] ×100, which equaled 19.1%. This indicated that sex and age importantly confounded the relationship between being dentate with caries and CKD outcome. In the other group of edentulous patients, the adjusted PR estimates of 1.67 and 1.69 obtained from Models 2 and 4 were markedly different from the crude PR estimate of 2.31 in Model 1. The greatest magnitude of confounding was determined when Model 1 was compared to Model 2 by the calculation of [(2.31-1.67)/1.67] ×100, which equaled 38.3%. Sex and age were therefore indicated as important confounders for the relationship between being edentulous and CKD outcome. In contrast, a small percent difference between Models 1 and 3 was obtained from [(2.31-2.27)/2.27] ×100, which equaled 1.8%. This indicated that BMI did not significantly change the relationship between edentulism and CKD outcome. Since the confounding was present, the measure of association between the main exposure of dentate status and dental caries and CKD outcome should rely on the estimates in Model 4 to obtain de-confounded effect estimates (Table 3).

**Table 3:** Multivariable analyses for the role of dentate status and presence of dental caries on the outcome of chronic kidney disease

Variables	Model 1: Unadjusted model (crude model)		Model 2: Adjusted for sex and age		Model 3: Adjusted for body mass index		Model 4:Adjusted for all covariates	
	PR (95% CI)	*P* value	PR (95% CI)	*P* value	PR (95% CI)	*P* value	PR (95% CI)	*P* value
**Main exposure**								
**Dentate status and dental caries**								
Dentate and caries-free	Reference		Reference		Reference		Reference	
Dentate with caries	1.12 (0.53, 2.37)	0.764	0.94 (0.45, 1.98)	0.882	1.12 (0.53, 2.37)	0.765	0.94 (0.45, 1.98)	0.880
Edentulous	2.31 (0.90, 5.91)	0.081	1.67 (0.63, 4.40)	0.298	2.27 (0.88, 5.82)	0.088	1.69 (0.64, 4.44)	0.290
**Covariates**								
**Sex**								
Female			Reference				Reference	
Male			2.13 (1.23, 3.71)	0.007			2.16 (1.24, 3.79)	0.007
**Age group**								
< 60 years			Reference				Reference	
≥ 60 years			3.52 (1.37, 9.03)	0.009			3.45 (1.32, 9.06)	0.012
**BMI**								
18.5 – 24.9 (Normal weight)					Reference		Reference	
< 18.5 (Underweight)					0.72 (0.18, 2.93)	0.646	0.88 (0.23, 3.46)	0.859
25.0 – 29.9 (Overweight)					0.77 (0.40, 1.48)	0.440	0.81 (0.43, 1.55)	0.527
≥ 30 (Obese)					0.83 (0.38, 1.81)	0.648	0.99 (0.46, 2.13)	0.974

PR, Prevalence ratio; CI, Confidence interval

All models were estimated by Poisson regression with robust standard errors.

**Table 4:** Multivariable analyses for the role of dentate status and presence of dental caries on the outcome of diabetic foot complication

Variables	Model 1: Unadjusted model (crude model)		Model 2: Adjusted for sex and age		Model 3: Adjusted for hypertension [HT]		Model 4: Adjusted for body mass index [BMI]		Model 5: Adjusted for both BMI and HT		Model 6: Adjusted for all covariates	
	PR (95% CI)	*P* value	PR (95% CI)	*P* value	PR (95% CI)	*P* value	PR (95% CI)	*P* value	PR (95% CI)	*P* value	PR (95% CI)	*P* value
**Main exposure**												
**Dentate status and dental caries**												
Dentate and caries-free	Reference		Reference		Reference		Reference		Reference		Reference	
Dentate with caries	1.11 (0.80, 1.52)	0.534	1.10 (0.80, 1.51)	0.567	1.10 (0.80, 1.51)	0.577	1.12 (0.82, 1.54)	0.480	1.11 (0.81, 1.52)	0.525	1.10 (0.80, 1.51)	0.551
Edentulous	1.32 (0.83, 2.09)	0.240	1.26 (0.78, 2.03)	0.346	1.29 (0.81, 2.04)	0.284	1.37 (0.86, 2.19)	0.190	1.33 (0.83, 2.12)	0.233	1.27 (0.79, 2.04)	0.331
**Covariates**												
**Sex**												
Female			Reference								Reference	
Male			0.97 (0.75, 1.26)	0.842							0.97 (0.75, 1.25)	0.805
**Age group**												
< 60 years			Reference								Reference	
≥ 60 years			1.14 (0.86, 1.51)	0.372							1.15 (0.86, 1.54)	0.332
**Hypertension**												
No					Reference				Reference		Reference	
Yes					1.70 (0.82, 3.51)	0.150			1.68 (0.81, 3.47)	0.165	1.63 (0.78, 3.38)	0.192
**BMI**												
18.5 – 24.9 (Normal weight)							Reference		Reference		Reference	
< 18.5 (Underweight)							0.69 (0.34, 1.38)	0.293	0.68 (0.34, 1.36)	0.276	0.68 (0.34, 1.38)	0.288
25.0 – 29.9 (Overweight)							0.93 (0.69, 1.24)	0.614	0.90 (0.67, 1.21)	0.486	0.93 (0.69, 1.25)	0.611
≥ 30 (Obese)							1.19 (0.87, 1.61)	0.274	1.14 (0.84, 1.56)	0.401	1.19 (0.86, 1.64)	0.291

PR, Prevalence ratio; CI, Confidence interval

Multivariable models were estimated by Poisson regression with robust standard error.

For the diabetic foot complication outcome (Table 4), PR estimates across all models ranged from 1.10 to 1.12 for the dentate with caries group and from 1.26 to 1.37 for the edentulous group, with all *P* values being non-significant. In the dentate with caries group, none of the adjusted PR estimates from Models 2 to 6 noticeably differed from the crude PR estimate of 1.11 in Model 1. The percent changes between the crude PR and adjusted PR estimates in all models were less than 1%, indicating that none of the covariates acted as important confounders. Nonetheless, PR estimates close to 1 in all models indicated no relationship between being dentate with dental caries and diabetic foot complications.

In the edentulous group, adjusted PR estimates from Models 2 to 6 also did not significantly differ from the crude PR estimate of 1.32 in Model 1. The greatest percentage difference was observed between Models 1 and 2 from [(1.32-1.26)/1.26] ×100, equaling 4.8%. All other percentage differences across models were below the 10% threshold, indicating that none of the covariates were important confounders for the relationship between being edentulous and diabetic foot outcome. Given the lack of evidence for confounding, the association between dentate status and diabetic foot complication can primarily be interpreted using the crude PR estimates from Model 1. Alternatively, the PR estimates from Model 6 could also be considered if controlling for residual confounding were ensured (Table 4).

## DISCUSSION

Unlike studies employing a sampling approach to select a population subset, this hospital-based study used a population-based approach to collect service-based data from all diabetic patients attending care during the designated study period. This inclusive method, without exclusion criteria, enabled a comprehensive description of patient characteristics and the estimation of the real-world prevalence and burden of diabetic complications and dental conditions within this district hospital setting.

Although the sex-specific prevalences of diabetes mellitus in Thailand only marginally differed between women (10.8%) and men (8.9%) [19], a significant disparity was observed in this study, with 62.8% of patients seeking diabetic care during the study period being women. The markedly greater proportion of female diabetic patients utilizing health services might be due to a higher likelihood of reporting symptoms, more health awareness, and greater concern for potential diabetic complications [20,21].

The average age of patients in this study was 63.6 years, with 67.8% aged 60 years or older, indicating that the study population predominantly consisted of elderly individuals. Nonetheless, the younger portion of patients in this study included individuals as young as 35 years old. This finding aligns with trends observed in the Thai population [19].

A high prevalence of dental caries (70.1%) was observed among patients in this study. This finding aligns with a previous study, which reported a similar caries prevalence of 73.3% among patients with diabetes using the same hospital-based study design [22]. The prevalence of edentulism among diabetic patients in this study was 8.2%, which was comparable to the 8.7% prevalence among the general Thai population between the ages of 60 and 75 [23]. The findings of these oral conditions could serve as evidence that oral healthcare must be an integral part of comprehensive care for patients with diabetes.

Among the three diabetic complications examined in this study, diabetic foot complication was the most prevalent (37.1%), followed by CKD (10.1%) and diabetic retinopathy (1.4%). The prevalence of diabetic foot complications was comparable to the 40% reported in a previous study of Thai diabetic patients. However, the prevalence of CKD and diabetic retinopathy in this study was substantially lower than the 48.2% and 31.2%, respectively, reported in that study [24]. This disparity may be attributed to differences in service contexts: the current study was conducted in a district-level secondary care hospital, whereas the previous study was conducted in a tertiary care teaching hospital, which serves as a referral center for more complicated diabetic cases [24].

Evidence from this study indicates no significant relationship between dental caries and CKD among patients with diabetes. This conclusion is supported by the inconclusive direction of PR estimates, ranging from 0.94 to 1.12 across all regression models, the proximity of PR estimates to 1 (the null value), and non-significant *P* values (Table 3). Similarly, regarding the relationship between dental caries and diabetic foot complication, although PR estimates across all regression models ranged from 1.10 to 1.12, suggesting a consistent direction that dental caries increased the prevalence of diabetic foot complications, the magnitude of all PR estimates was close to 1, and non-significant *P* values were obtained (Table 4). These findings collectively suggested no meaningful relationship between dental caries and diabetic foot complications. Although it has been hypothesized that dental caries could contribute to systemic inflammation [7], potentially exacerbating glycemic control [8,9] and increasing the risk of diabetic complications, the results of this study do not support this hypothesis.

Edentulism may be a more relevant factor in the higher prevalence of CKD and diabetic foot complications among diabetic patients, as suggested by the direction and magnitude of PR values in this study, even though none of the estimates reached statistical significance. PR estimates across all regression models ranged from 1.67 to 2.31 for the relationship between edentulism and CKD. These values were consistently further from the null PR value of 1 and demonstrated a uniform direction, suggesting that edentulism is associated with an increased prevalence of CKD (Table 3). Similarly, for diabetic foot complications, PR estimates ranged from 1.26 to 1.37, showing a slight but consistent deviation from the null value and suggesting that edentulism also increased the prevalence of this outcome (Table 4). The role of edentulism as an oral comorbidity of diabetes adversely affecting general health has been previously suggested [12]. Additionally, the direction of the relationship suggesting that edentulism might increase the prevalence of CKD and foot complications among patients with diabetes in this study was in line with the findings in another study that similarly compared three groups of diabetic patients and illustrated that glycemic levels and odds of hyperglycemia were significantly greater in the edentulous group than the two other groups with lower levels of periodontitis [15]. That study also explained that edentulism could represent the severe consequence of periodontitis, which is strongly associated with diabetes mellitus [15]. The evidence from this and previous studies supports the hypothesis that edentulism may be associated with CKD and diabetic foot complications among patients with diabetes, which should be further investigated using more rigorous epidemiological study designs such as cohort studies.

### Strengths and limitations

Key strengths of this study included the data collection from the entire patient population in the study setting, the application of DAG, and the careful application of statistical techniques for precise PR estimation. By analyzing service-based data from the entire diabetic patient population, the study captured the real-world burden of diabetic care, providing valuable insights for service improvement within this context. The use of DAG enabled rational identification of the roles of explanatory variables, especially the potential confounders to be controlled in regression analysis. The application of Poisson regression with robust standard errors was preferred to logistic regression in this study as it could provide more accurate estimates of exposure-outcome relationships, and the obtained PR was simpler to understand than the odds ratio [25].

Nonetheless, the cross-sectional study design was limited in evaluating the temporal relationship between the exposure and outcome of interest [26]. Therefore, the findings of the relationship in this study should be used for rationally generating hypotheses to be further tested by more rigorous epidemiological designs [26] rather than being interpreted as the final evaluation of the association. Additional factors that may influence the relationship between oral conditions and diabetic complications—such as the duration of diabetes, types of diabetic treatments, and compliance with treatment regimens—should also be considered in future studies. Furthermore, studies conducted in larger hospital settings, such as tertiary care or teaching hospitals, would enable the collection of more diabetic retinopathy cases, facilitating a more robust assessment of the relationship between oral conditions and diabetic complications.

## CONCLUSION

Although not statistically significant, the magnitude and direction of PR estimates suggest a potential hypothesis that, among patients with T2DM, edentulism may be associated with a higher prevalence of CKD and diabetic foot complications compared to being dentate and caries-free. These findings provide a rationale for hypothesizing that edentulism could play a role in these complications and provide fundamental knowledge, especially the use of directed acyclic graphs that future studies may consider when testing the hypothesis using more rigorous epidemiological study designs.

## Personal thanks

The authors would like to acknowledge the director and personnel of Prachathipat Hospital, who provided permission and technical support for using the service-based data for this research.
